# Dataset and validation of the approaches to study skills inventory for students

**DOI:** 10.1038/s41597-021-00943-6

**Published:** 2021-06-24

**Authors:** Skarlatos G. Dedos, Dimitris Fouskakis

**Affiliations:** 1 Department of Biology, National and Kapodistrian University of Athens, Panepistimioupoli Zografou, Athens, 15784 Greece; 2 Department of Mathematics, National Technical University of Athens, Zografou Campus, Athens, 15780 Greece

**Keywords:** Education, Education

## Abstract

There exists a vast amount of research on how students, in higher education, approach their studying and learning. Such research resulted in a multitude of questionnaires and tools to capture the way students perform in higher education institutions. One of these tools is the Approaches to Study Skills Inventory for Students (ASSIST) that was developed in the ’80 s and ’90 s. This inventory broadly classifies students, as approaching their study, in a deep, a strategic and/or a surface manner. Although it has gone through rigorous validation in many studies, there exist no publicly available dataset of the results of these studies and so the raw datasets cannot be re-used or integrated with other similar datasets. Here, we report and make publicly available the raw data of an 8-year longitudinal survey using this inventory in a cohort study of 1181 students from a department of a higher education institution. We validated our dataset using reliability analyses that confirmed, and compared well, with the results of previous studies.

## Abstract (summary)

**Table:** 

Measurement(s)	Study • learning
Technology Type(s)	questionnaire
Factor Type(s)	temporal interval • age • gender • parental education level
Sample Characteristic - Organism	Homo sapiens
Sample Characteristic - Location	Athens

Machine-accessible metadata file describing the reported data: 10.6084/m9.figshare.14665848

## Background & Summary

How can we enhance students’ studying in higher education? This multi-faceted question has been the focus of a great number of studies^1–12^ and it is generally regarded that the students’ involvement with the curriculum is one of the most decisive factors. Several tools have been developed to help academic teachers assess students’ approaches to study, such as the Vermunt’s inventory of Learning Styles in Higher Education (ILS)^13^, the Approaches to Study Skills Inventory for Students (ASSIST)^14^, the Kolb’s Learning Style Inventory (LSI)^15^, the Visual, Aural, Read/Write, Kinaesthetic learning-styles inventory^16^, or the Motivated Strategies for Learning Questionnaire (MSLQ)^17^, among many others^10–12,18^. One line of research, dating back to the ‘70 s focused on the approaches to studying and through a repeated improvement of an original questionnaire, arrived into a 52 items inventory that came to be called Approaches to Study Skills Inventory for Students^14,19^. The responses to this 5-point Likert scale inventory, when analysed as groups of 4 items (questions) clustered into sub-scales and scales, provide a broad attribute to the responder as having a deep approach, a strategic approach or a surface approach to studying^14^. Several weaknesses, as well as strengths, have been identified in this inventory^11^, one among them being the labelling of individuals, rather than behaviours, as being “deep” or “strategic” or “surface” learners^11^. Although one study^20^ identified the absence of clear polarity between the three approaches in subsets of students, several studies have validated this inventory in diverse higher education institutions in many countries.

Here, we report and make publicly available the results of a multi-sample longitudinal study that asked undergraduate students to complete, in real time, a Greek version of the inventory^21^. In the first year of the study (2011), students were not asked to provide their names and departmental ID so as to avoid any refusal to participate. From the 2^nd^ year (2012) and until the 8^th^ year (2018) of this study, students were asked to provide their departmental ID and name if they wished, and most of the students happily did. No names are included and the departmental IDs of the students are codified in the publicly available dataset^22^. For each year, we sought to have the same students completing the inventory so as to identify whether there are any changes in their responses. We have then integrated the responses of the students into a single file^22^. Specific information about the inventory and the data collection procedure are provided in the Methods Section.

One of the broader goals, that motivated us to collect the data, was the absence of any publicly available dataset from any study that has used this inventory so that we could make an in-depth validation and compare any analyses done on other datasets vis-à-vis our dataset. By having the collected dataset publicly available we believe that we increase the potential re-use value of the inventory and enable further analysis and further comparative research to be carried out on the way undergraduate students manage their studies. Another broader goal was the need to identify students who have a “surface” approach to studying^23,24^. Such students have been documented in many other studies^2,7,9,14,19^ as having an ineffective approach to studying by simply memorising facts just to pass a course, an approach that is ill-suited in today’s tertiary education where a vast amount of information is available^6,23^. By making this dataset public, we hope we can enable other researchers to quickly identify such students by analysing their responses to the inventory and thereby redesign, wherever appropriate, instructions and assessment.

## Methods

### Participants

Tertiary education in Greece is public and exclusively provided by higher education institutions that are subject to state supervision and financed by the government. Admission of students to these higher education institutions depends on their performance at nation-wide exams that take place yearly when students complete their secondary education (mostly at the age of 18). Enrolment to the various schools or departments of the Greek universities depends on the general score obtained by the secondary education graduates at these nation-wide exams, on the number of available places at each higher education institution and on the candidates’ ranked preferences among schools and departments of a university. Enrolment to a school or department of a university entails the attendance of the departmental program of study that lasts for 8 semesters (4 years) in most cases. Therefore, enrolment to a school or department of a university in Greece is defined as the registration, by obtaining a departmental ID, of a student to only one departmental program of study. Students who complete their studies are awarded their degree when they have passed the necessary number of courses stipulated in the departmental program and have accumulated the required number of credits.

All 1181 participants had enrolled in the Department of Biology, National and Kapodistrian University of Athens since 2007 (with 5 exceptions^22^). The Department of Biology enrolled on average 161,4 students/year in the 10-year cohort (2007–2016) of participating students (Table 1). The survey was carried out for 8 consecutive years, from 2011 until 2018. Participation in the study is defined as the number of students that have completed and handed in the inventory every time they were asked, starting on the spring semester of 2011 when students attending all 4 years of the departmental program (i.e. those with a departmental ID from 2007 to 2010) were asked to complete the inventory and lasting until the spring semester of 2018 for students that enrolled in the Department of Biology until 2016 (Table 1). Participation in the study was calculated to be 72.37% (N = 1614) if all students are accounted for, and 86.97% (N = 1297), if the students that dropped out of the program (N = 317) are excluded (Table 1). A total of 2029 hard copies of the inventory were collected in this longitudinal study from students ranging from the 1^st^ to the 5^th^ year of study (Table 1). For 81 students, we managed to have their responses for each year of the 4-year program and for 221 and 192 students we managed to have their responses for the two years and three years, respectively, of the 4-year program. The Greek version of the inventory^21^ was handed to each student before the beginning of practical/laboratory classes of variable courses, early in the spring semester (2^nd^ semester of each academic year). Students were asked to provide their departmental ID and/or their name if they wished. In the first year of the survey (2011) students were not asked to provide their departmental ID or their name so as to determine whether they will complete the inventory without the fear of being identified. As it turns out from the rates of participation in the 8-year survey, students displayed a high rate of voluntary participation (Table 1).

**Table 1:** Tabulation of the number of students that enrolled at the Department of Biology relative to those that responded to the Approaches to Study Skills Inventory for Students (ASSIST).

Academic Year^1^	Student Cohort	Enrolled	Graduated	Non-Graduates^4^	Discontinued their Studies^5^	Participants’ Year of Study (1^st^/2^nd^/3^rd^/4^th^ year)
	Survey Participants					
2007	Cohort^2^	158	91	40	27	(N.D./N.D./N.D./76)
	Participants^3^	48%				
2008	Cohort	155	93	33	29	(N.D./N.D./116/N.D.)
	Participants	75%				
2009	Cohort	144	86	35	23	(N.D./78/1/8 + 3*)
	Participants	63%	12%	6%		
2010	Cohort	153	88	36	29	(76/1/25/5 + 4*)
	Participants	73%	40%	14%		
2011	Cohort	142	77	32	33	(52/29/10/16 + 3*)
	Participants	77%	70%^6^	38%^6^	6%^6^	
2012	Cohort	153	76	34	43	(99/5/2/1)
	Participants	70%	100%	71%	16%	
2013	Cohort	188	80	76	22	(74/64/11/5)
	Participants	82%	100%	87%	36%	
2014	Cohort	201	21	134	46	(150/5/ N.D./N.D.)
	Participants	77%	100%	89%	33%	
2015	Cohort	159	N.D.	133	26	(125/4/N.D./N.D.)
	Participants	81%		94%	15%	
2016	Cohort	161	N.D.	122	39	(120/N.D./N.D./N.D.)
	Participants	75%		95%	10%	
Total	Cohort	1614	612	675	317	(696/186/165//111 + 10*)
	Participants	72%	45%	70%	13%	

The survey lasted for 8 years (2011–2018) therefore, N.D.: No Data collected. The numbers in brackets indicate the number of students that participated in the survey per year and category and % indicates their percentage relative to the cohort shown in the same cell. ^1^The year that the students enrolled to the undergraduate program of the Department of Biology and acquired their unique departmental ID, ^2^Total number of students that enrolled at the 4-year study program each year, ^3^Percentage of students that responded to the survey at least once, ^4^Number of students that have not yet graduated as of 08/2019, ^5^Number of students that have dropped out of the program as of 08/2019, ^6^42 students that did not report their departmental ID, *Number of students that deferred enrolment to the program for 1 year. There are 8 additional students that could not be assigned to a specific academic year due to incomplete demographic information they provided and 5 students with reported deferral of enrolment to the program for more than 1 year.

### Questionnaire

The English version of the Approaches to Study Skills Inventory for Students (ASSIST)^14,19,25–27^ was used as presented^19^. It was translated into Greek^21^ by the authors and was compared to an earlier Greek version of the inventory^28^ aiming to translate the meaning of each statement rather than provide an accurate translation of each statement. The Greek version was then back translated to English and was further cross-examined for the accuracy of each statement.

The English version of the inventory^19^ consists of three parts. All three parts of the inventory consist of a 5-point Likert scale where the highest number (i.e. 5) indicates agreement with the item (question) being asked and therefore places the responder as scoring high in this item. Conversely, the lowest number (i.e. 1) indicates disagreement with the item (question) being asked and therefore places the responder as scoring low in this item. The first part of the inventory contains 6 items (questions) about the conceptions of learning that have not been thoroughly analysed in the relevant literature^14,25^.

The second part of the English version contains 52 items (questions). This part has been mostly used on its own in the relevant literature, it is generally referred as the Revised ASI or RASI^14,29^, and generates scores on the Deep Approach, the Strategic Approach and the Surface Approach to studying (Tables 2–4)^14,25,26,30^.

**Table 2:** Structure of the second part of the Approaches to Study Skills Inventory for Students.

Scales (Approaches)	Sub-scales	Items
**Deep**	Seeking meaning (SM)	4, 17, 30 and 43
	Relating ideas (RI)	1, 21, 33 and 46
	Use of evidence (UE)	9, 23, 36 and 49
	Interest in ideas (II)	13, 26, 39 and 52
**Strategic**	Organised studying (OS)	1, 14, 27 and 40
	Time management (TM)	5, 18, 31 and 44
	Alertness to assessment (AA)	2, 15, 28 and 41
	Achieving (AC)	10, 24, 37 and 50
	Monitoring effectiveness (ME)	7, 20, 34 and 47
**Surface**	Lack of purpose (LP)	7, 20, 34 and 47
	Unrelated memorising (UM)	6, 19, 32 and 45
	Syllabus-boundness (SB)	12, 25, 38 and 51
	Fear of failure (FF)	8, 22, 35 and 48

Each sub-scale is formed by the sum of the responses to four particular items (questions) of the second part of the inventory. In turn, each of the three identified^14,19^ approaches (Scales) to studying is formed by the sum of the responses to the items (questions) that comprise the four (or five in the case of the Strategic Approach) sub-scales.

**Table 3:** Cronbach’s α reliability index for the sub-scales and the Deep, Strategic and Surface Approach scales.

Deep Approach	0.838	Strategic Approach	0.844	Surface Approach	0.731
	(N = 1914)		(N = 1950)		(N = 1946)
Seeking meaning	0.525	Organised studying	0.568	Lack of purpose	0.527
	(N = 1985)		(N = 1998)		(N = 2005)
Relating ideas	0.660	Time management	0.751	Unrelated memorising	0.465
	(N = 1985)		(N = 2016)		(N = 1994)
Use of evidence	0.579	Alertness to assessment	0.615	Syllabus-boundness	0.530
	(N = 1999)		(N = 2009)		(N = 1997)
Interest in ideas	0.650	Achieving	0.630	Fear of failure	0.640
	(N = 2005)		(N = 2008)		(N = 2018)
		Monitoring effectiveness	0.573		
			(N = 2011)		

For each Cronbach’s *α* value the sample size is also shown.

**Table 4:** Cronbach’s α reliability index for the sub-scales and the Deep, Strategic and Surface Approach scales calculated in this study and previous studies.

Scales and sub-scales	Greece^22,31^	Portugal^36^	Portugal^35^	Norway^33^	Ireland^37^	USA^37^	Ireland^34^
	(this study) N = 2029	N = 386	N = 566	N = 573	N = 437	N = 298	N = 286
**Deep Approach**	**0.838**	**0.78**	**0.81**	**0.81**	**0.84**	**0.82**	**0.81**
Seeking meaning	0.525	0.51	0.51	0.49	0.63	0.55	0.66
Relating ideas	0.660	0.47	0.54	0.62	0.59	0.59	0.55
Use of evidence	0.579	0.59	0.59	0.49	0.59	0.49	0.63
Interest in ideas	0.650	0.59	0.56	0.64	0.69	0.67	0.54
**Strategic Approach**	**0.827**	**0.81**	**0.83**	**0.81**	**0.87**	**0.87**	**0.86**
Organised studying	0.568	0.55	0.51	0.59	0.63	0.55	0.54
Time management	0.751	0.63	0.65	0.72	0.74	0.77	0.76
Alertness to assessment	0.615	0.47	0.40	0.41	0.63	0.56	0.67
Achieving	0.630	0.58	0.67	0.66	0.68	0.63	0.65
Monitoring effectiveness	0.573	0.55	0.58	0.51	0.61	0.61	0.52
**Surface Approach**	**0.731**	**0.66**	**0.79**	**0.70**	**0.83**	**0.80**	**0.80**
Lack of purpose	0.527	0.59	0.54	0.68	0.75	0.68	0.73
Unrelated memorising	0.465	0.48	0.73	0.57	0.59	0.57	0.53
Syllabus-boundness	0.530	0.47	0.62	0.57	0.64	0.55	0.61
Fear of failure	0.640	0.57	0.63	0.57	0.74	0.72	0.77

For each Cronbach’s *α* value the sample size is also shown.

In more detail, the sum of responses to the 5-point Likert scale of particular items form a particular sub-scale (see Table 2)^14,19^, while the sum of responses to the 5-point Likert scale of 16 or 20 items (questions) (Table 2) form the Deep, the Strategic and the Surface Approach to studying^14,19^. When a responder scores high on the sum of the responses to the 5-point Likert scale of, for example, the 16 items that form the Deep Approach to studying, then, depending on the analysis, the responder can be categorised as exhibiting a “deep” approach to studying^11,14,19,20^.

The third part contains 8 items (questions) about the students’ preferences for different kinds of teaching. It is also composed of a 5-point Likert scale as described above and this part has also not been thoroughly analysed in the relevant literature^14,25^. The inventory also contains a self-rating question presented as a 9-point Likert scale in which students are asked to assess their own academic progress starting from 1 as “rather badly” and ending to 9 as “very well”^14,19,21^.

The Greek version of the inventory^21^, that we asked students to complete, also contained, on its first page, questions about their parents’ education background (i.e. whether their father and/or mother have completed primary, secondary or tertiary education), and questions about their gender, age and year of study^21,22^.

### Data collection and integration

Hard copies of the inventory were collected by one of the authors, handed to another person to tabulate them into an Excel file and then cross-examined for accuracy of each entry. Then, the departmental ID of each student was given a random codified number to provide anonymity. A consecutive response number (i.e. 1, 2, 3 or 4) was given if multiple responses from each student were collected^22^. Each responder’s gender was codified as 1 = male or 2 = female. Their age was left as stated by each responder (≥18)^22^ and their year of study (termed year of study at assessment)^22^ was left as stated by each responder (≥1)^22^. The dataset was enriched with information about the year that each responder completed the inventory and the year that they enrolled at the Department of Biology^22^. Missing values were left intact. All data were then transferred into an SPSS v26 (Armonk, N.Y., IBM Corp) file for analysis.

## Data Records

The Data Records consist of 4 separate files that have been deposited to Figshare data repository system.

The first Data Record (10.6084/m9.figshare.13507341.v4) is the Greek version of the Approaches to Study Skills Inventory for Students and it is deposited for validation and re-use purposes, containing both the Greek text and the original text in English from which the translation was generated^21^.

The second Data Record (10.6084/m9.figshare.13507362.v4) is an Excel file that contains all the responses to the inventory and additional information for each responder^22^. Details about the contents of each column of the dataset are given on its first row and a codebook, in.htm format, accompanies this record in which details about the percentages of missing values and the responses are documented^22^.

The third Data Record (10.6084/m9.figshare.13507374.v2) is an SPSS output file, in.htm format, that contains all the dataset’s validation results, with the most important ones presented in Tables 3 and 4. This record contains all the reliability analysis tests and the inter-item Spearman correlation matrix results^31^.

The fourth Data Record (10.6084/m9.figshare.13507383.v2) is an Excel file that contains a description of the courses taught in the Department of Biology during the 4 years of undergraduate study and during the years that the survey took place. Data collection was terminated in 2018^32^, after the Department implemented a new undergraduate program in 2017, however records about the students’ completion of the program were collected until August 2019. Details about the contents of each column of the dataset are given on its first row of the spreadsheet^32^.

## Technical Validation

**Fig. 1:**
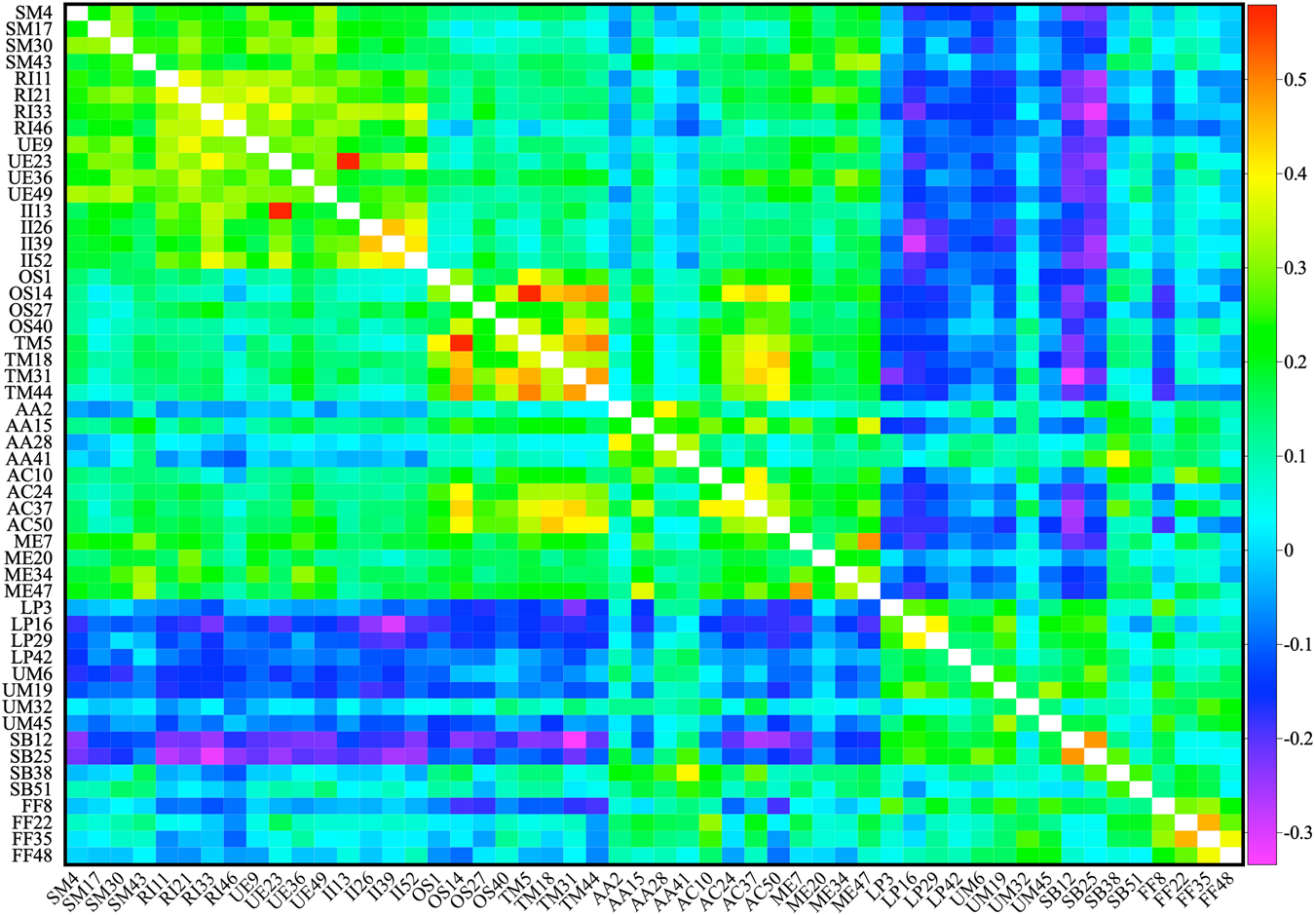
Inter-item Spearman correlation matrix for the items in the Approaches to Study Skills Inventory for Students. The 52 items of the second part of the inventory are ordered so that the first 16 (SM, RI, UE and II) form the Deep Approach scale, the next 20 (OS, TM, AA, AC and ME) form the Strategic Approach scale and the last 16 (LP, UM, SB and FF) form the Surface Approach scale as described^14,19^. Numbers indicate the order of the appearance of the items in the inventory. The Spearman correlation matrix was generated using SPSS v26 without correcting for missing values. Abbreviations are: SM, Seeking Meaning. RI, Relating Ideas. UE, Use of Evidence. II, Interest in Ideas. OS, Organised studying. TM, Time Management. AA, Alertness to Assessment. AC, AChieving. ME, Monitoring Effectiveness. LP, Lack of Purpose. UM, Unrelated memorising. SB, Syllabus-Boundness. FF, Fear of Failure. The same abbreviations are used in^31^.

As done in all of the relevant literature^14,33–36^ of this inventory, technical validation consisted of reliability analysis on SPSS v26 using Cronbach’s *α*^14^ reliability index (SPSS > Analyse > Scale > Reliability analysis) (Tables 3 and 4) and inter-item Spearman correlation matrices (SPSS > Analyse > Correlate > Bivariate) (Fig. 1). The results in Fig. 1 show that, as suggested in many other studies^14,19,20,33,34^, the sub-scale “Monitoring effectiveness” displays ambivalent Spearman correlation coefficients with the sub-scales that are grouped in the Deep Approach and the Strategic Approach^14,20^, while the “Alertness to Assessment” sub-scale displays an anomalous Spearman correlation with the other sub-scales that form the Strategic approach, a result that has been reported before and resulted in its exclusion in a previous study^20^.

The results in Table 3 show the Cronbach’s *α*^14^ reliability index and the number of responses that the students gave in each of the sub-scales. Of note is the fact that the Cronbach’s *α*^14^ reliability index does not change much if the sub-scale “Monitoring effectiveness” is grouped within the other items of the Deep Approach (0.846 (N = 1904)^31^ vis-à-vis 0.838 (N = 1914); Table 3).

Finally, we tabulated (Table 4) the Cronbach’s *α*^14^ reliability index for each sub-scale and each approach against the results reported in previous studies^14,33–35,37^. The results in Table 4 show that the Cronbach’s *α* reliability index falls within and does not exceed any of the previously reported values within the sub-scales and the three different scales (approaches) that the inventory identifies. Notable exception is the sub-scale “Unrelated memorising” which is below all the values shown in Table 4.

## Usage Notes

To the best of our knowledge, this is the first report that makes publicly available a dataset that is related to the Approaches to Study Skills Inventory for Students. This gives other researchers the opportunity to re-analyse the dataset in multiple ways. For example, other researchers can re-analyse the dataset by re-arranging or omitting^20^ items of each sub-scale to improve the reliability of the analysis. Alternatively, other researchers can opt to regroup the items in a way that will unmask other covert approaches to studying^23^. As a third and very important example, our dataset can be used to analyse the first part and the third part of the inventory that have not been thoroughly analysed to date^14,25^ and can be a rich source of correlated information between the responses in the first or third part and the responses to the second part of the inventory. Moreover, other researchers that have worked with the same inventory in the past have now the opportunity to compare this dataset with those that may have been generated even several decades ago and thus identify any inter-generational aspect(s) of how students’ approach to studying may or may not have changed.

By making publicly available the dataset we generated, we also hope that other researchers will use this dataset in cross-cultural and interdisciplinary studies. Due to its multidimensional nature, containing responses to the inventory and other metrices without identifying each student, the dataset can be integrated with datasets that others may generate in the future.

## Data Availability

The IBM® SPSS® software v26, licensed to the National and Kapodistrian University of Athens (http://www.cc.uoa.gr/texniki-ypostiri3h/egkatastash-paketwn-logismikoy/spss.html), was used for the dataset analysis^31^ with no specific variables or parameters used to process the current dataset. The Microsoft® Excel for Mac Version 16.16.14 (190909) was used to tabulate the responses to the inventory and generate the dataset^22^ before analysis with the IBM® SPSS® software v26. No custom code was used in the generation or processing of the dataset. The codebook that accompanies the second Data Record was generated by the IBM® SPSS® v26 software (SPSS > Analyse > Reports > Codebook).
